# Cross-species mapping of psychedelic gene expression reveals links to the 5HT2A receptor, cortical layers, and human accelerated regions

**DOI:** 10.21203/rs.3.rs-7625999/v1

**Published:** 2025-10-03

**Authors:** Lorenzo Pasquini, Patrick McConnell, Jackson Raffety, Andrew Li, Eric Steinberg, Syed Rahim, Christian Valtierra, Adam Gazzaley, Robin Carhart-Harris

**Affiliations:** UCSF, San Francisco; UCSF, San Francisco; UCSF, San Francisco; UCSF, San Francisco; UCSF, San Francisco; UCSF, San Francisco; UCSF, San Francisco; University of California at San Francisco

**Keywords:** Gene expression, human accelerated regions, neuroimaging, psychedelics, serotonin

## Abstract

Psychedelic drugs exert rapid and profound effects on human consciousness and are increasingly explored for their clinical potential. Yet, the genetic programs through which psychedelics reshape brain function and structure remain incompletely understood, in part because most studies have been conducted in preclinical models and cell cultures. We conducted a systematic literature search of transcriptomic studies in animal models and cell cultures to identify genes changing expression within 5 hours from the administration of a classical psychedelic. By cross-referencing with the Allen Human Brain Atlas, we identified a set of high-confidence psychedelic-responsive genes expressed in the human brain. These genes showed selective enrichment in cortical pyramidal neurons (layers 5 and 6) and were associated with Gene Ontology categories linked to neuron projection and neuronal spine morphology. Strikingly, psychedelic-responsive genes were overrepresented among human accelerated genes, suggesting an evolutionary dimension to their regulation. Spatial expression of the gene set was selectively correlated with the cortical distribution of the 5HT2A receptor, the canonical target of classical psychedelic compounds. Clustering analysis further revealed three distinct cortical gene expression modules, potentially reflecting separable biological pathways engaged by psychedelic action in the human brain. Together, these findings delineate a convergent molecular architecture through which psychedelics may shape cortical circuits and provide a translational framework to link cellular gene expression changes with macroscale neurobiology in humans.

## Introduction

Serotonergic psychedelics such as psilocybin, lysergic acid diethylamide (LSD), and N,N-dimethyltryptamine (DMT), have re-emerged as compounds of major interest in psychiatry and neuroscience due to their profound effects on the brain affecting perception, mood, and cognition (1–6), with multiple trials demonstrating fast and sustained clinical improvements across various neuropsychiatric disorders (7–13). A growing body of studies exploring the neural underpinnings of psychedelics, provides evidence that psychedelics engage neuroplasticity-related processes in the mammalian brain, including dendritic growth, synaptogenesis, and transcriptional reprogramming (14–18). Indeed, transcriptomic studies in animal models have revealed widespread changes in gene expression following psychedelic administration, implicating pathways relevant to neuronal signaling and plasticity (19, 20). Despite recent advances mapping psychedelic-associated genes into the human brain (21–23), it remains unclear how these preclinical genetic signatures relate to the human brain, where the therapeutic and experiential effects of psychedelics ultimately manifest.

At the receptor level, classical psychedelics primarily act as agonists of the serotonin 2A (5HT2A) receptor (24). In the human cortex, the 5HT2A receptor is predominantly expressed by excitatory pyramidal neurons in layers 5 and 6, particularly within higher-order associative cortices (25–27), which have been strongly implicated in both the subjective and therapeutic effects of psychedelics (25–27). Importantly, these same areas have undergone marked evolutionary expansion in humans (28, 29), with studies showing that human accelerated regions (HARs)—short stretches of non-coding DNA showing rapid evolutionary change in humans (30)—are preferentially expressed in areas overlapping with brain regions expressing the 5HT2A receptor (28, 29, 31, 32). This suggests a potential link between psychedelic drug action, cortical circuits underlying higher cognition, and genes shaped by recent evolution.

To further elucidate this translational link, we integrate gene expression data from preclinical psychedelic studies with the Allen Human Brain Atlas and evolutionary gene sets. We first conduct a systematic literature search to identify genes changing expression within 5 hours from the administration of a classical psychedelic. Having identified these genes, we ask: (1) whether psychedelic-responsive genes map onto particular neuron-types, cellular processes, and human accelerated genes; (2) whether they colocalize with the distribution of the 5HT2A receptor and other serotonergic receptors; and (3) how their spatial expression varies across cortical regions. Our approach enables us to investigate the relevance of psychedelic-induced transcriptomic changes in animal models to the cellular, receptor, and network-level architecture of the human brain. By bridging molecular pharmacology with human brain organization, this work provides a framework for understanding the pathways through which psychedelics may act in the human cortex.

## Methods

### Literature search of brain genes changing expression with psychedelic administration.

From 01/01/2025 to 05/30/2025, P.A.M., J.R., A.L., L.P., E.S., S.R., and C.V. conducted a systematic literature search to identify articles describing changes in gene expression with the administration of a classical psychedelic (**Supplementary Figure S1**, **Supplementary Table S1**, and **Supplementary Appendix S1**). The search and related data extraction followed PRISMA guidelines, documenting metadata such as tissue type, analysis method, and findings. All references were imported into Covidence, where duplicates were identified and removed. The articles underwent single-reviewer screening based on title and abstract, with a refined PICOS model of systematic review. The search was conducted on Pubmed and Google scholar using search terms including: “gene expression” AND “psychedelic”, “transcription” AND “psychedelic”, “expression” AND “psychedelic” (see **Supplementary Appendix S1**).

Identified articles were screened to meet following inclusion criteria: (*i*) be peer reviewed original research articles (no preprints, reviews, or secondary analyses); (*ii*) used a classical serotonergic psychedelic (including LSD, psilocybin, psilocin, and dimethoxyiodoamphetamine [Doi]). Studies focusing on non-classic psychedelics (e.g., ketamine, ibogaine, MDMA, cannabis) were excluded; (*iii*) describe changes in gene expression in human or animal brain tissue or neural cell culture. Plants, fungi, insects, artificial intelligence models, and computational simulations were excluded. Non-neuronal studies or peripheral tissues without clear links to brain/neuronal functions were excluded; (*iv*) have made the gene lists publicly available; (*v*) use an eligible drug administration with a placebo (between-subjects) or baseline (within-subjects) control (i.e., expression significantly increased or decreased relative to the control). Studies with no eligible drug administration or control condition were excluded. Studies with no significant eligible findings or studies with findings specifically related to preclinical translational model (e.g., addiction and stress pre-conditioning models, knockout models) or pre-treatment condition (e.g., inhibitor) were excluded (33); (*vi)* assess changes in expression either under acute psychedelic administration or in the days to weeks following one-to-two administrations of a psychedelic. Following this search (14, 34–47), only genes acutely changing expression under a classical psychedelic (defined as within 5h from dosing) were further analyzed in this study.

### Human expressed genes from the Allen Human Brain Atlas.

Average human brain gene expression data were derived from microarray data available through the Allen Human Brain Atlas (AHBA) (http://human.brain-map.org/static/download). As described in detail elsewhere (48), tissue samples were extracted across both hemispheres from two human brain donors, as well as the left hemisphere of four additional donors, totaling 3,702 tissue samples. Microarray analysis quantified gene expression across 58,692 probes, providing an estimate of the relative expression of 20,734 genes within the tissue samples (49). The publicly available toolbox abagen (https://github.com/rmarkello/abagen)(50) was then used for: *(i)* updating probe-to-gene annotations using the latest available data; *(ii)* data filtering, where expression values that do not exceed background are removed; *(iii)* probe selection, which, for genes indexed by multiple probes, involves selecting a single representative measure to represent the expression of that gene across all donor brains; *(iv)* sample assignment, where tissue samples from the AHBA were mapped to 200 parcels of the Schaeffer brain atlas. The Schaeffer atlas was selected since it has been widely used in genetic neuroimaging studies and functional MRI studies in general (51). Its popularity is driven by the fact that it has been derived through a data-driven parcellation algorithm applied to functional brain connectivity data, yielding approximatively homologous bilateral regions, with each region being assigned to one out of seven primary intrinsic brain networks (51), including the visual, somatosensory, dorsal attention, ventral attention, frontoparietal, default mode, and limbic networks (51). Steps *i-iv* were followed by *(v)* normalization of expression measures to account for inter-individual differences and outlying values; *(vi)* gene-set filtering, to remove genes that are inconsistently expressed across the six brains; and *(vii)* averaging gene expression across donors. This procedure resulted in the average expression of 15,634 genes (**Supplementary Figure S2**) being mapped on 189 out of 200 cortical brain parcels, with 11 parcels missing expression values across all genes (**Supplementary Table S2**). Due to substantial differences in gene expression values when comparing subcortical and cortical parcels (52), no subcortical atlas was used in this study. See **Supplementary Appendix S2** for a more detailed report on the procedures using abagen.

### Gene ontology and cell enrichment analyses.

Over the course of the study, we assessed the overlap between genes derived from the AHBA dataset and genes changing expression under acute (within 5 hours) classical psychedelic administration in animal models. This procedure yielded a set of 51 genes acutely changing expression under psychedelics (GCEP) with a high confidence of being also expressed in the human brain (referred to as GCEP_AHBA_ from now on). The list of GCEP_AHBA_ were entered into a gene ontology analysis using the publicly available software EnrichR (https://maayanlab.cloud/Enrichr/) (53) which was queried for gene ontology cellular components significantly associated with the set of GCEP_AHBA_. EnrichR is an open-source software platform for visualizing molecular interaction networks and biological pathways and integrating these networks with annotations, gene expression profiles, and other state data. Additionally, a cell enrichment analysis was conducted using a publicly available cell-type specific enrichment analysis (CSEA) software (http://doughertytools.wustl.edu/CSEAtool.html) (54). CSEA is bioinformatics method for identifying which cell types are most enriched for a given gene set. Benjamini-Hochberg corrected *p* values are reported for both the gene ontology and cell-enrichment analysis, unless specified otherwise.

### Human Accelerated Regions (HAR) containing genes.

HAR containing genes were taken from a comparative genomic analysis that identified, from a list of several recent publications, loci with accelerated divergence in humans when compared to chimpanzees (30). A total of 2,737 HARs were identified, representing 2,164 unique HAR-associated genes (30). Of these 2,164 HAR genes, 1,373 were identified as sufficiently expressed in the brain based on the AHBA brain-expressed gene dataset (probes used if they exceeded background signal for more than 50% of all samples) and used in our analyses, referred to simply as HAR_AHBA_ genes(28) (**Supplementary Data**). The overlap of HAR_AHBA_ genes and GCEP_AHBA_ was assessed through a one-tailed Fisher’s exact tests using the *fisher.test* function in R (https://www.r-project.org/) (*p* < 0.05). For this test, the totality of AHBA genes were used as background.

### Correlation of GCEP_AHBA_ maps and regional expression of the 5HT2A receptor.

Regional brain expression values for the 5HT2A receptor were derived from the AHBA dataset. These regional expression estimates of the 5HT2A receptor were correlated against expression maps of GCEP_AHBA_, yielding a distribution of correlation values reflecting the similarity in regional expression between the 5HT2A receptor and identified GCEP_AHBA_. A random set of 51 non-GCEP were derived from the AHBA dataset, and their maps were correlated with regional expression estimates of the 5HT2A receptor. The resulting distribution of correlation values was compared with correlation values derived using GCEP_AHBA_ using a two-sample *t*-test (*p* < 0.05). Furthermore, we used a permutation-based test to compare the average correlation of GCEP_AHBA_ to the average correlations of non-GCEP_AHBA_. 51 non-GCEP_AHBA_ were randomly selected 5,000 times from the AHBA. For each permutation, the average correlation between maps of non-GCEP_AHBA_ and the 5HT2A receptor were calculated, resulting in a distribution reflecting the similarity between randomly selected non-GCEP_AHBA_ and the 5HT2A expression maps. We then derived a *p* value by counting how often the average random correlation was equal or higher than the empirical average correlation between GCEP_AHBA_ and the 5HT2A receptor. Maps of the 5HT1A, 5HT2A, 5HT2C, and Dopamine 1 (DR1) receptors, as well as of the serotonin transported (HTT), were also derived from the AHBA atlas and correlated with maps of GCEP_AHBA_. An ANOVA and associated post-hoc t-test (FDR corrected *p* < 0.05) were used to assess differences in correlation between GCEP_AHBA_ maps and the different receptor maps.

### Human co-expression network of GCEP_AHBA_.

The regional expression values of GCEP_AHBA_ were correlated with each other to generate a gene-to-gene regional co-expression (GCE) matrix. Graph theoretical approaches were applied to the resulting co-expression matrix to generate topographical network representations of gene expression (55). This matrix was binarized at a Pearson’s correlation coefficient threshold of 0.3 and the number of surviving edges between genes was counted for each gene of interest to derive a measure of nodal degree, reflecting the level of connectedness between a specific gene and each other gene selected for the analysis. Resulting data were rendered as a graph using the Brain Network Connectivity Toolbox (https://sites.google.com/site/bctnet/) in MATLAB (https://www.mathworks.com/products/matlab.html). The Louvain community detection algorithm implemented in the Brain Connectivity Toolbox was then used to identify communities within the CGE matrix, reflecting clusters of genes showing similar patterns of regional co-expression. Community detection was implemented using the resolution parameter *G* = 0.5, which allows for the detection of larger communities, and a symmetric treatment of negative weights. The cluster identity of each gene was then used to generate average maps of gene expression for each cluster.

### Human global functional connectivity on LSD.

We leveraged publicly available neuroimaging data from a published neuroimaging study assessing the impact of LSD on brain function (1). This data involved resting-state fMRI data acquired in healthy participants while under the effects of LSD (n = 15) or an inactive placebo (n = 15). Details on data preprocessing and acquisition can be found elsewhere (1). The fMRI data were parcellated into the Schaefer 200 space, and, for each individual, we derived functional connectivity matrices by correlating the BOLD time-series of each brain region. We then averaged this matrix across columns to derive a regional estimate of global functional connectivity (GFC) (56, 57). Individual estimates of GFC were averaged across the LSD and placebo groups and subtracted to derive a map reflecting GFC differences between the LSD and placebo conditions. We then used Pearson’s correlation to assess the spatial similarity between this LSD-placebo GFC difference map and maps of gene clusters identified in the previous analysis. Because spatial autocorrelation inherent to neuroimaging data can inflate *p*-values in brain map analyses (58), we corrected for this autocorrelation by applying a permutation-based approach, in which 5,000 surrogate maps that preserve the autocorrelation properties of the LSD-placebo GFC map were generated using the toolbox BrainSMASH (https://brainsmash.readthedocs.io/en/latest/approach.html). We then derived autocorrelation-corrected *p*-values by counting how often the correlation between surrogate maps and each gene cluster map was equal to or higher than the true correlation and divided this number by the total number of permutations (*p* < 0.05).

## Results

### Rat and mice genes acutely changing expression under classical psychedelics overlap with human brain expressed genes from the AHBA.

**Figure 1.**
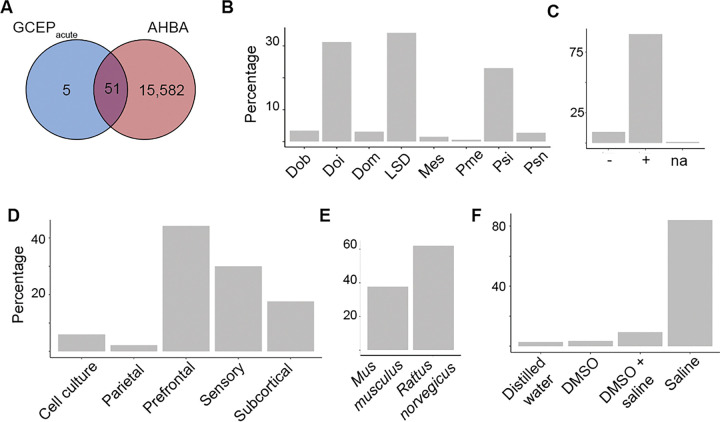
Genes changing expression under acute psychedelic administration overlap with human brain-expressed genes. **(A)** A literature search identified 56 unique genes changing expression in preclinical models under the acute administration (within 5h) of a classical serotonergic psychedelic. Of these 56 genes changing expression under psychedelics (GCEP_acute_), 51 overlapped with genes derived from the AHBA, having therefore a higher confidence of being expressed in the human brain (GCEP_AHBA_). **(B)** Most human GCEP showed increased expression (+), with a minority of genes showing decreases (−), both decreases and increases (−/+), or having inconclusive data (na). **(C)** Acute expression changes were primarily induced by Doi, LSD, or psilocybin. **(D)** Changes in expression were primarily assessed in the prefrontal cortex, primary sensory cortex, and subcortical areas. **(E)** Studies used the rat (*Rattus norvegicus*) or mice (*Mus musculus*) as the animal model of choice and **(F)** saline as a control. AHBA: Allen Human Brain Atlas; DMSO: Dimethyl sulfoxide; Dob: dimethoxybromoamphetamine; Doi: dimethoxyiodoamphetamine; LSD: lysergic acid diethylamide; Psi: psilocybin; Psn: psilocin; na: not available.

Out of the initial search (N = 474), 324 unique articles were abstract- and title-level screened in Covidence systematic review software. 77 articles were then subject to full-text review, yielding a subset of studies (n = 17) meeting the inclusion criteria, providing data on neural gene expression changes induced by classical psychedelics (**Supplementary Figure S1** and **Supplementary Table S1**). The search yielded 156 unique gene entries, of which 56 were genes changing expression within 5h from dosing (mean time [SD] = 1.5h [0.5h], max = 5h, min = 0.75h), referred to as from now on as acutely changing genes. Of these 56 genes, 51 overlapped with genes from the AHBA, increasing the confidence that these genes acutely changing expression under classical psychedelics (GCEP_AHBA_) are expressed also in the human brain (Fig. 1A, **Supplementary Table S3-S4**). Changes in GCEP_AHBA_ were primarily quantified under the administration of Doi, LSD, or psilocybin (Fig. 1B). Most genes were shown to be overexpressed (Fig. 1C), with a minority undergoing reduced expression when compared to a placebo. Gene expression levels were primarily quantified in prefrontal, sensory or subcortical areas (Fig. 1D) using mice and rat as the primary models (Fig. 1E) and saline as control (Fig. 1F).

### Human GCEPs are enriched for layer 5 and 6 neurons, neurite processes, and HAR genes.

**Figure 2.**
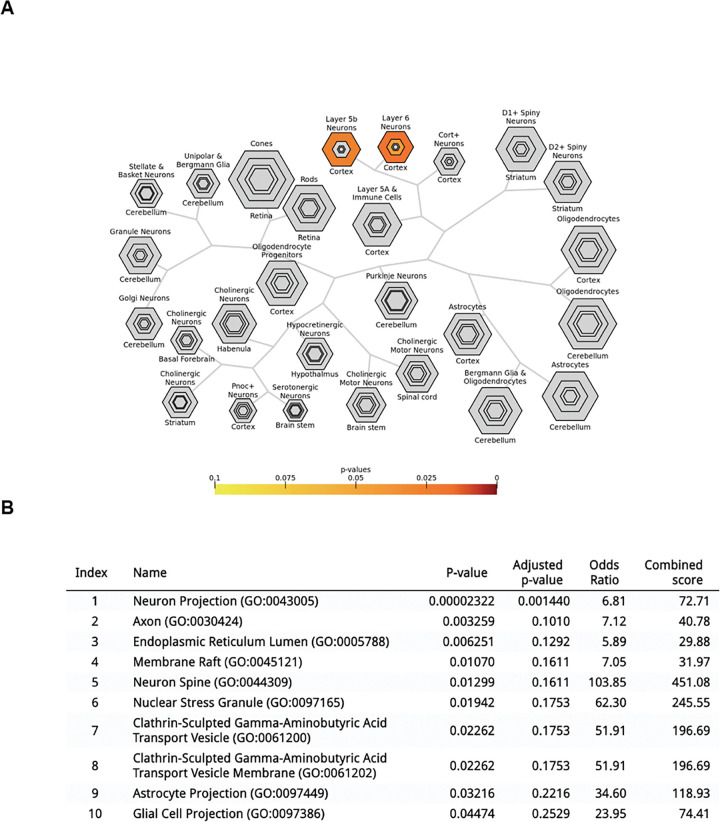
**(A)** A cell enrichment analysis leveraging the 51 GCEP_AHBA_ revealed a significant enrichment for layer 5b and layer 6 neurons. The bar shows Benjamini-Hochberg corrected *p*-values, which are used to color the hexagons, with less statistically significant p-values being reflected by the outermost hexagons. **(B)** A gene ontology analysis of cellular components associated with the 51 GCEP_AHBA_ revealed significant association with terms primarily linked to neuron projection (Benjamini-Hochberg corrected *p*<0.05), and with lower statistical confidence to glial projection, astrocyte projection, and neural spine (uncorrected *p*<0.05). GCEP_AHBA_: genes changing expression under psychedelics overlapping with human brain-expressed genes.

We next conducted a cell-enrichment analysis for the previously identified 51 GCEP_AHBA_ (Fig. 2A). This analysis revealed significant enrichments for cortical layer 5b and layer 6 neurons (Benjamini-Hochberg corrected *p* < 0.05). We next conducted a gene ontology analysis using the 51 GCEP_AHBA_ (Fig. 2B), revealing significant associations with cellular components associated with neuron projection (Benjamini-Hochberg corrected *p* < 0.05), and to a less significant degree to neural spine, as well as to axons, glial, and astrocyte projection (*p* < 0.05 uncorrected).

**Figure 3.**
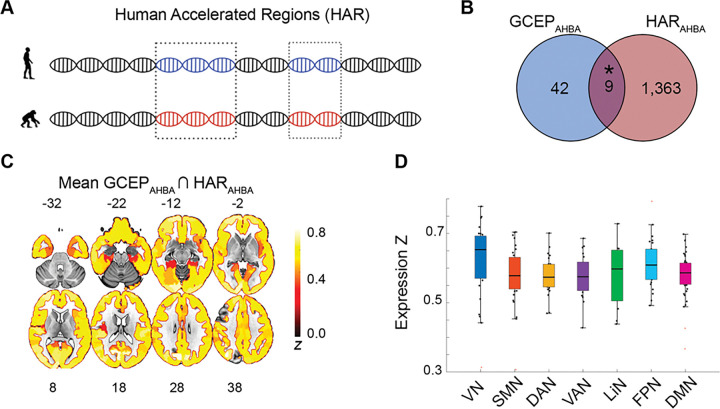
Overlap between human GCEP_AHBA_ and HAR genes. **(A)** HARs are conserved genomic loci that have undergone accelerated divergence in the human evolutionary lineage. **(B)** GCEP_AHBA_ had 9 genes in common with HAR genes expressed in the human brain. The overlap was significantly and unlikely to occur by chance when compared to a background set of human brain-expressed genes. **(C)** Average cortical expression of GCEP_AHB_ overlapping with HAR_AHBA_. **(D)** Average cortical expression of GCEP_AHB_ overlapping with HAR_AHBA_ plotted for canonical large-scale brain networks. HAR_AHBA_: genes containing human accelerated regions overlapping with human brain-expressed genes. DAN: dorsal attention network; DMN: default mode network; FPN: frontoparietal network; LiN: limbic network; SMN: Somatomotor network; VAN: ventral attention network; VN: visual network. *p<0.05. Panel **A** adapted with permission from(28,30).

Previous research has identified that humans brain regions changing activity under acute psychedelic administration overlap with brain regions showing recent cortical expansion and containing higher expressions of genes containing human accelerated regions (HAR genes; Fig. 3A). We leveraged a published list of HAR genes with a higher confidence of being expressed in the human brain (HAR_AHBA_), to show that these genes undergoing recent evolutionary pressure significantly overlap with GCEP_AHBA_ (*p* = 0.03; Fig. 3B, **Supplementary Table S3**). Some of these genes, like *LRRTM4* and *NEGR1* and have been involved in cell adhesion and regulation of synapse assembly (59, 60), while others, like *5HT2A*, *SLC17A6*, and *SST*, are important neuromodulators of the brain (31, 61), with *5HT2A* being primarily involved in the action of serotonergic psychedelics (24). The average expression map of HAR-associated GCEP_AHBA_ revealed that these genes, while ubiquitously expressed, show particularly dense expression within the visual, prefrontal, and medial and lateral parietal cortices (Fig. 3C–D).

### Maps of GCEP_AHBA_ correlate with the regional distribution of the 5HT2A receptor.

**Figure 4:**
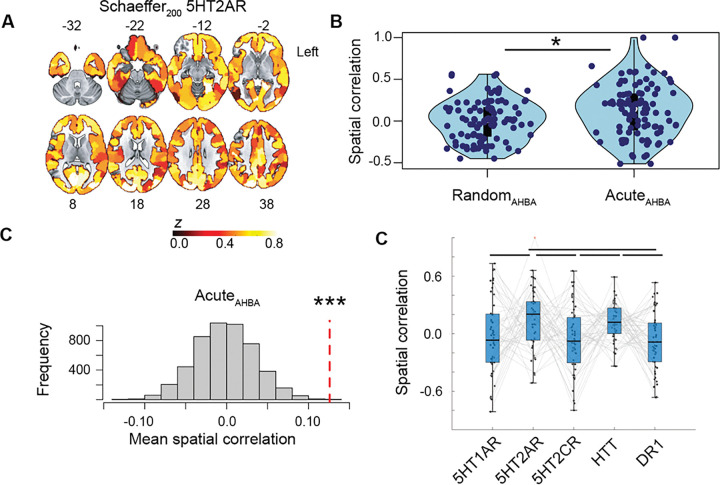
Spatial correlation between human GCEP and the 5HT2A receptor. **(A)** Regional cortical expression of the 5HT2A receptor estimated using the Schaeffer alas with 200 parcels. Warm colors reflect higher expression, while cold colors reflect lower expression. Color bar reflects z-scores of standardized gene expression. **(B)** Maps of GCEP_AHBA_ show stronger correlations to the regional distribution of the 5HT2A receptor when compared to non- GCEP_AHBA_ randomly selected from the AHBA. **(C)** Permutation test, yielding a distribution of average spatial correlations between the 5HT2A receptor and 51 non-GCEP_AHBA_ randomly selected 5,000 times from the AHBA. This distribution was compared to the average spatial correlations between the 5HT2A receptor and 51 GCEP_AHBA_ (vertical dotted red line). **(D)** Spatial correlation between GCEP_AHBA_ maps and spatial maps of the 5HT1A, 5HT2A, 5HT2C, and Dopamine 1 (DR1) receptors, as well as with the serotonin transporter (HTT). Lines above boxplots show post-hoc significant comparisons (Tukey-Kramer corrected *p*<0.0005. *p<0.0005; ***p<0.0005.

Given that serotonergic psychedelics act primarily on the 5HT2A receptor, we investigated whether the spatial distribution GCEP_AHBA_ in the brain resembled the expression of the 5HT2A receptor (Fig. 4A, **Supplementary Figure S2**). We leveraged regional expression maps of GCEP_AHBA_ and of randomly chosen non-GCEP_AHBA_, to show a significant higher spatial correlation to the 5HT2A receptor of GCEP_AHBA_ when compared to randomly chosen non-GCEP_AHBA_ (two-sample *t*-test, *t*(2,100) = 2.06, *p* = 0.04; Fig. 4B). A permutation test using 5,000 random resampling of non-GCEP_AHBA_, revealed a significant higher average correlation of GCEP_AHBA_ maps to the regional expression values of the 5HT2A receptor (mean-R(49) = 0.13, *p* = 0.0002; Fig. 4C). Having assessed that the GCEP_AHBA_ maps showed a stronger correlation to the spatial distribution of the 5HT2A receptor compared to randomly chosen genes, we next sought to assess whether this relationship was specific to the 5HT2A receptor by comparing the spatial correlation between GCEP_AHBA_ maps and the 5HT1A receptor, 5HT2C receptor, the dopamine receptor 1(DR1), and the serotonin transporter (HTT) (Fig. 4D). An ANOVA revealed a significant group effect (*F*(4,250) = 5.6, *p* = 0.0003), with post-hoc *t*-tests revealing increased similarity between GCEP_AHBA_ and both the 5HT2A receptor and HTT maps when compared to the 5HT1A, 5HT2C, or DR1 receptor maps (Tukey-Kramer corrected *p* < 0.05).

### Maps of GCEP_AHBA_ are differently expressed in the human brain.

**Figure 5.**
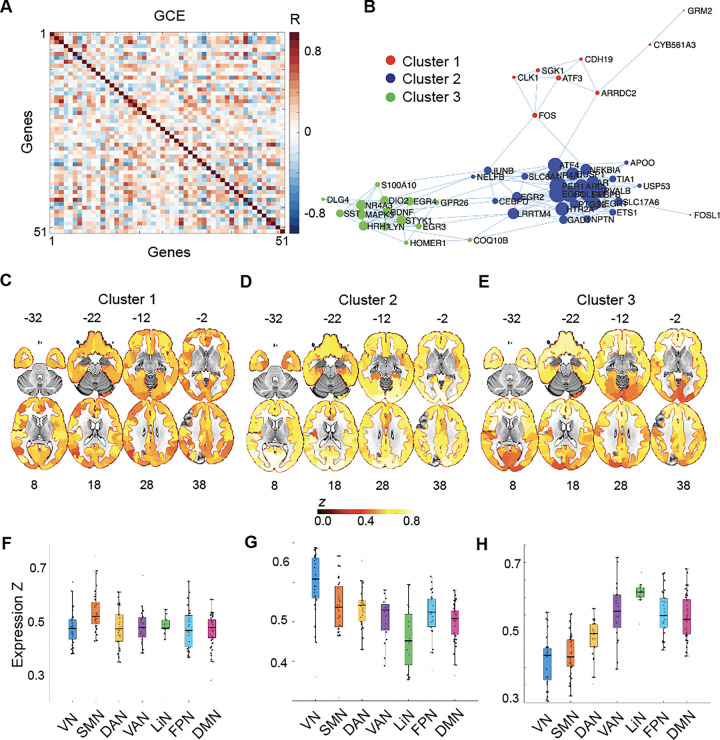
Anatomical gene expression clusters. **(A)** Gene co-expression (GCE) matrix reflecting how GCEP_AHBA_ are co-expressed across brain regions. Color bar reflects Pearson’s correlations; warm colors reflect positive, cold colors negative correlations. **(B)** The gene co-expression matrix was used to generate a topographical network representation of GCEP_AHBA_. The size of each circle reflects the centrality of each gene in the network, while the color of the circles reflects the assignment of each gene in three distinct clusters estimated through a community algorithm. Spatial maps of Cluster 1 (**C**), Cluster 2 (**D**) and Cluster 3 (**E**), reflecting how genes in each cluster are expressed in average across the human brain. Average expression of genes in Cluster 1 (**F**), Cluster 2 (**G**) and Cluster 3 (**H**) across human brain canonical large-scale brain networks. DAN: dorsal attention network; DMN: default mode network; FPN: frontoparietal network; LiN: limbic network; SMN: Somatomotor network; VAN: ventral attention network; VN: visual network.

We next used the regional expression of GCEP_AHBA_ across the human brain to derive a gene co-expression matrix, reflecting how similarly genes are expressed across brain parcels of the Schaeffer atlas (Fig. 5A). Using graph theoretical approaches, we then derived a topological network representation of GCEP_AHBA_, revealing three clusters of genes (Fig. 5B, **Supplementary Table S5**). These clusters of genes showed distinct patters of expression, either homogenously expressed in the brain (Cluster 1; e.g.,: *CDH19* and *FOS*; Fig. 5C and F), preferentially expressed in primary sensory cortices (Cluster 2; e.g.,: *LRRTM4* and *HT2A*; Fig. 5D and G), or preferentially expressed among higher cognitive brain areas overlapping with the prefrontal and temporal cortices (Cluster 3; e.g.,: *BDNF* and *SST*; Fig. 5E and H).

**Figure 6.**
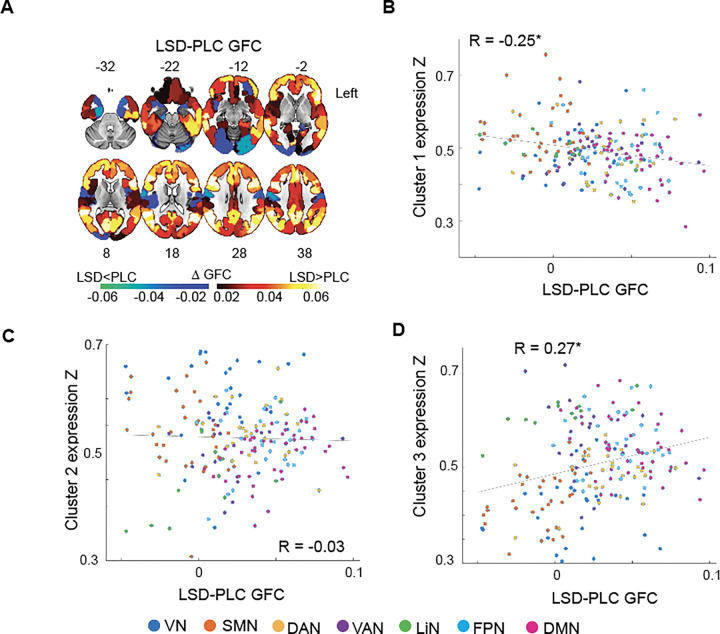
Global functional connectivity and gene expression clusters. **(A)** Spatial map reflecting changes in global functional connectivity (GFC) between the LSD and placebo (PCB) conditions. Red reflects increases in GFC for a specific region on LSD, while blue reflects decreases. Scatterplots reflect the spatial correlation between GFC changes on LSD and the maps of GCEP_AHBA_ Cluster 1 (**B**), Cluster 2 (**C**), and Cluster 3 (**D**). DAN: dorsal attention network; DMN: default mode network; FPN: frontoparietal network; LiN: limbic network; SMN: Somatomotor network; VAN: ventral attention network; VN: visual network. *p-permutation<0.05

To further investigate the biological significance of these gene clusters, we assessed the spatial similarity between the GCEP_AHBA_ cluster maps and functional brain changes induced by LSD when compared to placebo. We leveraged resting-state fMRI data from a published study (1) to derive regional estimates of global functional connectivity (GFC) (56, 57) – a measure reflecting how strongly the activity of each brain region is correlated to the activity of all other brain regions. We then assessed GFC differences between the LSD and placebo conditions, revealing that LSD increases GFC in prefrontal, medial, and lateral parietal areas, while GFC decreases on LSD were more prominent among the visual cortices (Fig. 6A). The LSD minus placebo GFC map correlated negatively with expression map of Cluster 1 (Fig. 6B, R(187) = −0.25, *p*-permutation = 0.040), did not significantly correlate with the expression map of Cluster 2 (Fig. 6C, R(187) = −0.03, *p*-permutation = 0.278), while correlated positively with the expression map of Cluster 3 (Fig. 6D, R(187) = 0.27, *p*-permutation = 0.048).

## Discussion

Our systematic literature search identified a set of genes changing expression following the administration of a classical psychedelic in preclinical models. From this gene list, a subset of genes changing expression within 5h from psychedelic administration overlap substantially with genes expressed in the human cortex. Importantly, many of these genes show upregulation after psychedelic administration, consistent with the view that psychedelics promote profound transcriptional changes, possibly underlying structural and functional changes induced by psychedelics. Through bioinformatic analyses, we demonstrate that psychedelic-responsive genes are enriched in cortical layer 5 and 6 neurons, large pyramidal cells that serve as principal cortical output channels, as well as enriched for terms related to spine formation and neural growth. These results resonate with prior work showing that psychedelics modulate excitatory output neurons and promote neuroplasticity effects (14–18, 25), particularly in prefrontal and association cortices, potentially underlying changes in brain function and structure (19,20,56). We further find that psychedelic-responsive genes are overrepresented among human accelerated genes (30), highlighting an intriguing evolutionary dimension. HARs are disproportionately expressed in higher-order association brain areas (28, 29, 31, 32), which are strongly modulated during the acute action of psychedelic substances (25–27). Our findings suggest that psychedelics may act on transcriptional programs that are not only central to cortical circuit function but also evolutionarily expanded in humans, potentially contributing to their unique capacity to alter consciousness (21–23).

A critical observation is that the spatial distribution of psychedelic-responsive genes aligns with that of the 5HT2A receptor, the canonical molecular target of classical psychedelics (25–27). This concordance provides mechanistic grounding for linking transcriptomic changes observed in animal models to receptor-mediated effects in the human cortex. Beyond the 5HT2A receptor, we also observe associations with other serotonergic receptors, primarily the serotonin transporter HTT, suggesting a broader serotonergic gene–receptor axis through which psychedelics may exert their acute effects (24). Clustering analyses of the regional expression patterns reveal that psychedelic-responsive genes segregate into three distinct cortical modules, each with a characteristic spatial expression profile, either (a) being evenly distributed, (b) centered on primary sensory cortices, or (c) on heterometal brain areas. Two out of three gene clusters spatially resembled large-scale functional brain changes induced by LSD (1). These gene clusters may correspond to separable biological pathways or network-level targets (62), raising the possibility that different facets of the psychedelic experience—from sensory alterations to emotional modulation to profound ego-dissolving states (63–65)—are linked to distinct gene sets (21–23).

Several limitations warrant consideration. First, the gene lists were derived from heterogeneous preclinical studies, with variation in species, compounds, doses, and time points. While the applied systematic literature search mitigates this limitation, the impact of the heterogeneity of study designs warrants further investigation. Second, the AHBA is derived from postmortem tissue data from a limited adult sample of six subjects, of which only three provided bulk transcriptomic data for both hemispheres. This limitation may not capture the full diversity of human cortical transcriptomics and may therefore hamper the generalizability of our findings. Further, our study focused on cortical gene expression, omitting subcortical areas despite the relevance of the thalamus and other subcortical areas in the acute action of psychedelics (25, 27, 66, 67). Gene expression levels from bulk tissue microarray dramatically differ depending on whether these are extracted from cortical or subcortical areas, with most studies, including ours, analyzing either one set of structures or the other (28, 29, 31). Third, our analyses are correlative in nature and cannot establish causal relationships between psychedelic administration, gene expression changes, and the genetic architecture of the human brain. Our analyses exclusively focused on genes changing expression within 5h of administration. Future studies could take a more nuanced look and explore gene expression changes within more fine-grained time bins, as well as explore long-lasting gene expression changes taking place after 5h. Despite these limitations, our work provides a translational framework linking psychedelic-induced transcriptomic changes to the cellular, receptor, and evolutionary architecture of the human cortex. By demonstrating enrichment in cortical output neurons, overlap with human accelerated genes, and alignment with the regional distribution of the 5HT2A receptor, we highlight convergent pathways through which psychedelics may shape human brain function and structure. These findings advance our understanding of the molecular and circuit-level substrates of psychedelics and provide testable hypotheses for future experimental and clinical studies.

## Supplementary Material

Supplementary Files

This is a list of supplementary files associated with this preprint. Click to download.

• SupplDataMcConnelletalCLEAN.xlsx

• GenpsyLPv1.2sm.docx

## Data Availability

Genes and associated information identified through the systematic search are available as ***Supplementary Data***.

## Significance Statement

This work bridges molecular pharmacology, systems neuroscience, and human evolution. By linking psychedelic-induced transcriptomic changes with human cortical cell types, receptor expression, and evolutionary gene programs, we provide a framework for understanding how psychedelics engage specific cortical circuits. This translational bridge advances mechanistic models of psychedelic action and may inform therapeutic development.
